# Sex Differences in the Association Between Disease Complications and Markers of Oxidative Stress in Type 2 Diabetes Mellitus: A Cross‐Sectional Study

**DOI:** 10.1002/hsr2.71734

**Published:** 2026-01-12

**Authors:** Titus Naa Yeng, Simon Bannison Bani, Moses Banyeh, Nafiu Amidu, Yussif Adams, P. P. M. Dapare, Albert Vendoor

**Affiliations:** ^1^ Department of Biomedical Laboratory Science, School of Allied Health Sciences University for Development Studies Tamale Ghana; ^2^ Laboratory Department Tamale Teaching Hospital Tamale Ghana; ^3^ Department of Clinical Chemistry, School of Allied Health Sciences University for Development Studies Tamale Ghana

**Keywords:** diabetic ketoacidosis, diabetic neuropathies, diabetic retinopathies, Ghana, malondialdehyde, sex differences, type 2 diabetes mellitus

## Abstract

**Background and Aims:**

Type 2 diabetes mellitus (T2DM) is often linked to oxidative stress resulting from lipid peroxidation and complications such as diabetic retinopathy, neuropathy, nephropathy, and ketoacidosis (DKA), which may manifest differently based on sex. However, the specific relationship between diabetic complications and markers of oxidative stress in relation to sex remains uncertain. This study seeks to elucidate sex‐based variances in the association between oxidative stress markers and diabetic complications among individuals with T2DM in the Ghanaian population.

**Methods:**

This study was conducted between July and December 2022 at the Tamale Teaching Hospital. The study involved 55 T2DM patients, comprising: no complications = 13; DKA = 13; neuropathy = 6; nephropathy = 14; and retinopathy = 9. The patients were aged between 20 and 55 years. Single fasting (8–10 h) venous blood samples were collected and analyzed for total cholesterol (TCHOL), high‐density lipoprotein cholesterol (HDL‐C), low‐density lipoprotein cholesterol (LDL‐C), malondialdehyde (MDA), and glutathione peroxidase (GSH‐Px) using an automated biochemistry analyzer and ELISA technique.

**Results:**

While no significant sex differences were found in complication prevalence, notable disparities emerged in glycemic and oxidative stress markers. Males with DKA exhibited higher MDA levels compared to females, while females with retinopathy had lower MDA levels than males. Additionally, males with DKA showed higher GSH_Px levels than females, while no significant differences were observed between sexes without complications, and males with DKA and retinopathy tended to have lower HDL‐C levels compared to females.

**Conclusion:**

While no significant differences in complication prevalence were observed between sexes, our findings revealed notable sex disparities in glycemic and oxidative stress markers. These results indicate the importance of considering sex‐specific factors in understanding the pathogenesis and management of diabetic complications.

## 1 Introduction

Diabetes mellitus remains a paramount global health challenge, intricately linked to a myriad of complications that profoundly affect afflicted individuals, encompassing neuropathy, nephropathy, and retinopathy [1, 2]. At the core of its pathophysiology lies oxidative stress, delineating an imbalance between the generation of reactive oxygen species (ROS) and the body's antioxidant defense mechanisms [3].

A pivotal mechanism within oxidative stress is lipid peroxidation, entailing the oxidative breakdown of polyunsaturated fatty acids [4]. This biochemical cascade yields detrimental byproducts such as malondialdehyde (MDA) and lipid peroxides, which significantly contribute to tissue damage and the progression of diabetic complications [4, 5]. Notably, oxidative stress, especially arising from lipid peroxidation, has been implicated in heightened diabetic mortality rates and the onset of life‐threatening complications such as retinopathy, neuropathy, and nephropathy [3, 6].

Although diabetic ketoacidosis (DKA) is classically associated with type 1 diabetes, it is increasingly recognized as a serious, albeit less common, complication in patients with type 2 diabetes mellitus (T2DM). The pathogenesis of DKA in T2DM typically involves a relative insulin deficiency compounded by increased counter‐regulatory hormones during periods of stress, infection, or poor glycemic control [7, 8, 9]. Importantly, DKA is associated with heightened oxidative stress, endothelial dysfunction, and systemic inflammation, which may exacerbate tissue injury and influence lipid peroxidation pathways [10, 11, 12]. Given this, investigating DKA in the context of oxidative stress markers such as MDA and glutathione peroxidase (GSH‐Px) is essential to understanding its contribution to diabetes‐related morbidity, especially in resource‐limited settings where late presentations are common.

The exploration of sex‐specific disparities in lipid peroxidation and its interaction with diabetic complications assumes paramount significance. The influence of sex hormones, particularly estrogen, on oxidative stress and lipid metabolism suggests the likelihood of distinct susceptibilities to diabetic complications between males and females [7, 8] [13].

Comprehending disease mechanisms, delineating therapeutic targets, and optimizing patient outcomes hinge upon elucidating the role of lipid peroxidation in T2DM and its intricate relationship with sex‐specific disparities in diabetic complications. Such insights are instrumental in devising sex‐tailored interventions to enhance the management of T2DM, accounting for the nuanced complications and physiological idiosyncrasies of both genders [9].

While extant literature has probed into sex‐related variations in T2DM and its complications [7, 10] [14], investigations specifically delving into sex disparities in lipid peroxidation remain scarce, particularly within the Ghanaian context. Given the genetic and environmental diversities across populations, the manifestation of sex differences in lipid peroxidation might exhibit variability. Consequently, unraveling these nuances in a local demographic holds the potential to inform tailored therapeutic approaches aimed at ameliorating T2DM management outcomes.

## 2 Materials and Methods

### 2.1 Study Design and Settings

The study enrolled 55 individuals diagnosed with T2DM, categorized as follows: without complications (*n* = 13), DKA (*n* = 13), neuropathy (*n* = 6), nephropathy (*n* = 14), and retinopathy (*n* = 9). Participants were aged between 20 and 55 years. Data collection took place at the Tamale Teaching Hospital (TTH) during the period spanning July to December 2022. TTH functions as a tertiary‐level healthcare facility, serving as a referral center for the northern regions of Ghana. Moreover, patients from neighboring countries, such as Burkina Faso, may also receive specialized care at TTH. The hospital encompasses various departments and specialized units, including the Diabetic Clinic, where this study was conducted.

### 2.2 Participants and Selection

Individuals diagnosed with type 2 diabetes, as defined by the WHO (2016) [2] and American Diabetes Association (2013) [15] [2, 11] guidelines with fasting plasma glucose (FPG) levels ≥ 7.0 mmol/L, were included in the study. These participants underwent standard clinical evaluation at the Diabetic Clinic of the TTH and were subsequently categorized into five groups based on their diabetic complications: without complications, diabetic neuropathy, diabetic retinopathy, diabetic nephropathy, and DKA. A total of 55 participants were enrolled in the study, comprising 30 females and 25 males.

For participants classified with DKA, blood samples were not collected during acute metabolic crises. Instead, samples were obtained during routine outpatient visits following documented clinical recovery. DKA classification was based on at least one confirmed episode within the past 6 months, as verified from hospital medical records.

Inclusion criteria encompassed individuals aged between 20 and 55 years, confirmed clinically to have type 2 diabetes. Exclusion criteria comprised individuals taking antioxidant supplements (e.g., folic acid and vitamin C) for a minimum of 1 month prior to the study, patients receiving dialysis or infusion therapy, pregnant women, and individuals with a smoking history of at least 1 year. For the *control group* (T2DM patients without complications), individuals were included if they had been diagnosed with type 2 diabetes for at least 1 year and had *no documented evidence* of neuropathy, retinopathy, nephropathy, or ketoacidosis, based on clinical history, physical examination, and relevant biochemical assessments. These participants met the same general inclusion criteria as other groups, including age (20–55 years) and absence of antioxidant supplementation, dialysis, pregnancy, or significant smoking history. This ensured comparability while serving as a baseline reference for evaluating the effect of diabetic complications on oxidative stress markers.

A smoker was defined as someone with a minimum of 6 months of regular cigarette smoking, while an ex‐smoker was someone who had ceased smoking for at least 1 year after regular smoking.

### 2.3 Sample Size Justification

Based on findings from a prior investigation, the mean ± SD (nmol/mL) serum level of MDA in diabetic patients with and without diabetic retinopathy was reported as 18.6 ± 3.67 and 12.0 ± 4.99, respectively [16]. Employing a statistical power of 0.80 at an *α* level of 5% (95% confidence interval [CI]), a power analysis for sample size determination was performed using G*Power version 3.1.9.7, which yielded a minimum requirement of 9 participants per group for a two‐tailed independent Student's *t*‐test, thus would be sufficient to detect statistically significant differences in oxidative stress markers. This threshold is supported by standard sample size estimation tables for Student's *t*‐test [17, 18]. Although retinopathy represented one of the smaller complication groups in our cohort, it provided the most robust published effect size for sample size estimation. This approach was chosen to ensure sufficient power in at least one clinically relevant subgroup. We acknowledge this as a limitation for other subgroups with fewer than ideal sample sizes and interpret their results cautiously, highlighting them as exploratory.

### 2.4 Data Collection and Measurement

Socio‐demographic information and participants' medical histories were gathered through structured pretested questionnaires. Venous blood samples were obtained from the antecubital vein following an overnight fast of 8–10 h. Five milliliters of blood were drawn, with one portion collected into a sodium fluoride vacutainer and the other into a clot activator/plain vacutainer tube. Blood samples in the clot activator tube were allowed to clot at room temperature before both tubes underwent centrifugation at 3000 × g for 10 min to separate serum or plasma [19, 20]. The resulting serum and plasma samples were then aliquoted and stored at −80°C until analysis, with strict adherence to the protocol of avoiding thawing and refreezing. All aliquots were analyzed within 2 weeks of storage to ensure biomarker integrity, particularly for GSH‐Px activity. Serum lipids were assessed using an automated biochemistry analyzer, while oxidative stress markers were measured via ELISA.

This sampling approach was designed to capture post‐acute, residual oxidative stress rather than transient fluctuations associated with acute DKA episodes. Evidence suggests that biomarkers of oxidative damage, including lipid peroxidation and DNA oxidation, may remain elevated for several weeks to months following DKA resolution [21, 22].

### 2.5 Statistical Analysis

The data were initially recorded in Microsoft Excel and subsequently analyzed using SPSS (version 27.0; IBM Corp., Armonk, New York) and GraphPad Prism (version 9.0; GraphPad Software, San Diego, California). The normality of quantitative variables was assessed using the Kolmogorov–Smirnov test. Normally distributed continuous data are presented as mean ± standard deviation (SD), while non‐normally distributed data are summarized as median and interquartile range (IQR). Categorical data are reported as frequencies and percentages.

Between‐group comparisons for continuous variables were conducted using independent samples *t*‐tests or one‐way analysis of variance (ANOVA) with Bonferroni post hoc correction, as appropriate. For non‐normally distributed variables, the Mann–Whitney *U* test or Kruskal–Wallis test was employed. Associations between categorical variables were evaluated using *χ* ^2^ tests or Fisher's exact tests.

Multinomial logistic regression was performed to estimate odds ratios (ORs) and adjusted odds ratios (aORs), with 95% CIs, while adjusting for potential confounders such as age and body mass index (BMI). Effect estimates and their precision are reported throughout.

All statistical tests were two‐tailed, and statistical significance was defined a priori as *p* < 0.05. No correction for multiple comparisons was applied, given the exploratory nature of subgroup analyses. There were no missing data. Statistical reporting and methodology were guided by the STROBE statement and the SAMPL guidelines.

### 2.6 Ethical Approval

Ethical approval for the study was obtained from the Institutional Ethics and Review Board of the University for Development Studies, Ghana (Approval Number: UDS/RB/047/22). All study procedures involving human participants adhered to the ethical principles of the 1964 Declaration of Helsinki and its subsequent amendments. Written informed consent was obtained from all participants before enrolment. Participant anonymity was maintained by de‐identifying data at the point of collection, and all personal information was handled in strict confidence and accessible only to the research team.

## 3 Results

### 3.1 The Socio‐Demographic Characteristics of the Study Population

The study population comprised predominantly female participants, representing 54% of the total sample. Smoking habits indicated that the majority of participants reported never smoked, representing 80% of participants (Table 1). Among the diabetic complications reported, diabetic nephropathy was the most prevalent (25.5%), while 23.6% of patients did not exhibit any diabetic complications (Table 1). Metformin was the most frequently reported medication among T2DM subjects (60.0%), followed by glimepiride (36.4%), with a small proportion of participants reporting no medication use (3.6%) (Table 1). The average BMI of the participants was 25.31 ± 4.78 kg/m^2^, consistent with the upper normal to overweight range (Table 1).

**Table 1:** The socio‐demographic characteristics of the study population.

Variable	T2DM (55)
Age (years)	47.16 ± 7.41 (95% CI: 45.16–49.16)
BMI (kg/m^2^)	25.31 ± 4.78 (95% CI: 24.02–26.60)
Sex	
Male	25 (45.5)
Female	30 (54.5)
Smoking	
Yes	0(0.0)
Never	44 (80.0)
Ex‐smoker	11 (20.0)
Drinking	
Yes	20 (36.4)
No	35 (63.6)
Diabetes complications	
None	13 (23.6)
Diabetes neuropathy	6 (10.9)
Diabetes retinopathy	9 (16.4)
Diabetes nephropathy	14 (25.5)
Diabetic ketoacidosis	13 (23.6)
Medication	
Metformin	33 (60.0)
Glimepiride	20 (36.4)
None	2 (3.6)

*Note:* Data are presented as mean ± standard deviation with 95% confidence intervals for continuous variables and as frequencies (percentages) for categorical variables.

Abbreviation: BMI = body mass index.

### 3.2 Association of Sex With Diabetic Complications

**Figure 1:**
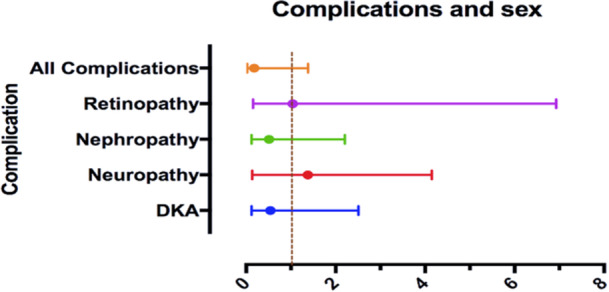
Multinomial logistic regression showing adjusted odds ratios (aORs, 95% CI) for diabetic complications in females, with males as the reference category. Results are presented as aOR: adjusted odds ratio (95% CI: confidence interval). aORs are adjusted for age and BMI.

The results, as shown in Table 2 and Figure 1, indicate that there were no statistically significant differences between males and females in the prevalence of diabetic complications. For DKA, 28% of males and 20% of females experienced this complication (*p* = 0.43, aOR = 0.543) (Table 2 and Figure 1). In terms of neuropathy, 16% of males and 6.7% of females were affected (*p* = 0.79, aOR = 1.379) (Table 2 and Figure 1). Similarly, for nephropathy, the rates were 32% in males and 20% in females (*p* = 0.37, aOR = 0.512), while for retinopathy, it was 8% in males and 23.3% in females (*p* = 0.97, aOR = 1.039) (Table 2 and Figure 1). Furthermore, the presence of one or more complications showed frequencies of 84% in males and 70% in females (*p* = 0.10, aOR = 0.177) (Table 2).

**Table 2:** Association of sex with diabetic complication: frequency and multinomial regression.

Sex	Complication status		aOR (95% CI)	*p* value
	Yes	No		
	DKA			
Male	7 (28%)	18 (72%)		
Female	6 (20%)	24 (80%)	0.543 (0.118–2.508)	0.43
	Neuropathy			
Male	4 (16%)	21 (84%)		
Female	2 (6.7%)	28 (93.3%)	1.379 (0.134–14.147)	0.79
	Nephropathy			
Male	8 (32%)	17 (68%)		
Female	6 (20%)	24 (80%)	0.512 (0.119–2.207)	0.37
	Retinopathy			
Male	2 (8%)	23 (92%)		
Female	7 (23.3%)	23 (76.7%)	1.039 (0.156–6.928)	0.99
	All complications			
Male	21 (84%)	4 (16%)		
Female	21 (70%)	9 (30%)	0.177 (0.023–1.385)	0.10

*Note:* Results are frequencies and multinomial regressions controlling for age and BMI with male as the reference group. Results are presented as frequency (percentage) and aOR: adjusted odds ratio (95% CI: confidence interval). *p* < 0.05 is considered statistically significant.

### 3.3 Glycemic and Oxidative Stress Indices of T2DM Subjects With and Without Complications Stratified by Sex

The investigation presented in Table 3 explored the interplay between glycemic and oxidative stress markers among individuals with T2DM, stratified by sex and the presence of complications. Notable patterns emerged across these subgroups.

**Table 3:** Glycemic and oxidative stress indices of type 2 diabetic subjects with and without complications stratified by sex.

Variable	Sex		Mean difference	95% CI	*p* value
	Male	Female			
	No complications				
FPG	10.70 ± 6.50	8.52 ± 4.55	2.18	−5.16 to 9.12	0.49
MDA	5.84 ± 1.11	5.40 ± 1.88	0.44	1.58 to 2.46	0.68
GSH‐Px	22.20 ± 4.51	19.12 ± 11.90	3.08	−8.69 to 14.85	0.63
	DKA				
FPG	12.17 ± 3.57	10.19 ± 5.20	1.98	−4.49 to 8.45	0.45
MDA	12.06 ± 6.30	5.77 ± 1.37	6.29	0.0 to 12.58	0.04*
GSH‐Px	25.26 ± 7.66	15.16 ± 1.97	10.1	2.38 to 17.82	0.01*
	Neuropathy				
FPG	12.29 ± 7.07	10.00 ± 6.75	2.29	−7.58 to 12.16	0.73
MDA	9.35 ± 7.55	5.71 ± 1.27	3.64	−3.82 to 11.1	0.56
GSH‐Px	16.21 ± 16.19	11.88 ± 0.97	4.33	−11.43 to 20.09	0.30
	Nephropathy				
FPG	10.19 ± 5.53	10.44 ± 4.67	−0.25	−6.94 to 6.44	0.93
MDA	7.21 ± 4.58	5.75 ± 1.72	1.46	−3.06 to 5.98	0.48
GSH‐Px	18.68 ± 5.04	14.66 ± 2.55	4.02	−1.2 to 9.24	0.10
	Retinopathy				
FPG	9.8 ± 0.42	8.02 ± 4.00	1.78	−17.8 to 21.36	0.57
MDA	9.27 ± 7.31	4.80 ± 0.85	4.47	−61.33 to 70.27	0.09
GSH‐Px	24.26 ± 1.47	15.55 ± 2.91	8.71	−10.52 to 27.94	0.01*
	All complications				
FPG	11.21 ± 4.91	9.52 ± 4.53	1.69	−1.35 to 4.73	0.25
MDA	9.43 ± 5.90	5.44 ± 1.31	3.99	1.24 to 6.74	0.004*
GSH‐Px	22.84 ± 8.80	14.84 ± 2.51	8.0	3.83 to 12.17	< 0.001*

*Note:* Results are presented as mean ± standard deviation (SD). Bivariate analysis was conducted using a two‐tailed unpaired *t*‐test to determine mean differences (male − female) with corresponding 95% confidence intervals (CIs). All comparisons were exploratory and unadjusted for multiple testing.

* Statistical significance at *p* < 0.05.

Across all complication categories, individuals with T2DM exhibited significantly elevated FPG levels relative to those without complications (*p* < 0.05) (Table 3). However, no significant sex‐based differences in FPG were observed within individual complication subgroups (Table 3).

Regarding oxidative stress markers, males with DKA had significantly higher levels of MDA compared to females (mean difference: 6.29 nmol/mL; 95% CI: 0.00 to 12.58; *p* = 0.04) (Table 3). In contrast, females with retinopathy exhibited lower MDA levels than males (mean difference: 4.47 nmol/mL; 95% CI: –61.33 to 70.27; *p* = 0.09) (Table 3). GSH‐Px was also significantly elevated in males with DKA compared to females (mean difference: 10.1 U/mL; 95% CI: 2.38 to 17.82; *p* = 0.01) (Table 3), although the difference in GSH‐Px levels between sexes for nephropathy did not reach statistical significance (mean difference: 4.02 U/mL; 95% CI: –1.20 to 9.24; *p* = 0.10) (Table 3).

Collectively, these findings suggest sex‐associated variation in oxidative stress responses among individuals with T2DM and specific complications. The observed differences, particularly in MDA and GSH‐Px levels among patients with DKA and retinopathy, may reflect underlying biological or hormonal influences on redox regulation. However, these results should be interpreted cautiously, and further mechanistic studies are warranted to elucidate potential sex‐based modifiers of oxidative stress in T2DM.

### 3.4 Fasting Lipid Indices of Type 2 Diabetic Subjects With and Without Complications Stratified by Sex

The findings depicted in Table 4 offer a comprehensive examination of fasting lipid indices among type 2 diabetic subjects, stratified by sex and the presence of complications. Our analysis reveals sex‐based trends in lipid profiles across these subgroups.

**Table 4:** Fasting lipid indices of type 2 diabetic subjects with and without complications stratified by sex.

Variable	Sex		Mean difference	95% CI	*p* value
	Male	Female			
	No complications				
TCHOL	6.29 ± 1.51	6.43 ± 1.37	−0.14	−1.57 to 1.29	0.87
TRIG	2.06 ± 1.11	1.57 ± 1.00	0.49	−0.94 to 1.92	0.45
HDL‐C	2.32 ± 0.65	2.34 ± 0.73	−0.02	−0.65 to 0.61	0.98
LDL‐C	0.96 ± 1.05	1.48 ± 1.01	−0.52	−0.65 to 0.61	0.42
VLDL	0.93 ± 0.50	0.72 ± 0.45	0.21	−0.37 to 0.79	0.44
	DKA				
TCHOL	5.78 ± 1.44	5.56 ± 2.04	0.22	−1.35 to 1.79	0.83
TRIG	1.37 ± 0.93	1.27 ± 0.92	0.10	−0.87 to 1.07	0.83
HDL‐C	1.68 ± 0.86	2.58 ± 0.86	−0.90	−1.90 to 0.10	0.08
LDL‐C	1.19 ± 1.09	1.89 ± 0.83	−0.70	−1.90 to 0.50	0.22
VLDL	0.62 ± 0.42	0.58 ± 0.42	0.04	−0.38 to 0.46	0.84
	Neuropathy				
TCHOL	5.72 ± 1.43	7.70 ± 2.11	−1.98	−5.36 to 1.40	0.23
TRIG	1.70 ± 0.49	1.83 ± 1.24	−0.13	−1.97 to 1.71	0.86
HDL‐C	2.25 ± 0.98	2.51 ± 1.64	−0.26	−2.09 to 1.57	0.81
LDL‐C	1.58 ± 1.25	1.50 ± 1.37	0.08	−1.78 to 1.94	0.96
VLDL	0.77 ± 0.22	0.83 ± 0.61	−0.06	−0.80 to 0.68	0.86
	Nephropathy				
TCHOL	5.81 ± 1.57	5.71 ± 0.86	0.10	−1.21 to 1.41	0.89
TRIG	1.68 ± 1.27	1.77 ± 0.84	−0.09	−1.22 to 1.04	0.88
HDL‐C	2.34 ± 0.91	2.09 ± 0.92	0.25	−0.79 to 1.29	0.63
LDL‐C	1.52 ± 0.78	1.37 ± 1.35	0.15	−1.03 to 1.33	0.80
VLDL	0.76 ± 0.57	1.05 ± 0.71	−0.29	−1.01 to 0.43	0.43
	Retinopathy				
TCHOL	6.42 ± 0.25	5.56 ± 1.30	0.86	−1.67 to 3.39	0.41
TRIG	0.87 ± 0.01	1.24 ± 0.61	−0.37	−1.33 to 0.59	0.45
HDL‐C	0.92 ± 0.13	2.35 ± 0.46	−1.43	−2.20 to 0.66	0.004*
LDL‐C	0.90 ± 0.18	1.67 ± 0.44	−0.77	−1.55 to 0.01	0.05
VLDL	0.40 ± 0.00	0.56 ± 0.28	−0.16	−0.66 to 0.34	0.46
	All complications				
TCHOL	5.84 ± 1.35	5.81 ± 1.53	0.03	−0.90 to 0.96	0.94
TRIG	1.50 ± 0.96	1.46 ± 0.81	0.04	−0.58 to 0.66	0.86
HDL‐C	1.97 ± 0.93	2.36 ± 0.78	−0.39	−0.92 to 0.14	0.15
LDL‐C	1.36 ± 0.93	1.63 ± 1.01	−0.27	−0.91 to 0.37	0.37
VLDL	0.68 ± 0.43	0.73 ± 0.51	−0.05	−0.44 to 0.34	0.76

*Note:* Results are presented as mean ± standard deviation (SD). Bivariate analysis was conducted using a two‐tailed unpaired *t*‐test to determine mean differences (male – female) with corresponding 95% confidence intervals (CIs). All comparisons were exploratory and unadjusted for multiple testing.

* Statistical significance at *p* < 0.05.

Among individuals without complications, no statistically significant differences were observed in total cholesterol (TCHOL), triglycerides (TRIG), high‐density lipoprotein cholesterol (HDL‐C), low‐density lipoprotein cholesterol (LDL‐C), or very low‐density lipoprotein (VLDL) between males and females (*p* > 0.05) (Table 4). For instance, HDL‐C levels were nearly identical (mean difference: –0.02 mmol/L; 95% CI: –0.65 to 0.61; *p* = 0.98) (Table 4), underscoring minimal sex influence in the absence of complications.

Conversely, notable disparities emerged in lipid indices among diabetic patients with complications. In DKA, males showed lower HDL‐C levels than females (mean difference: −0.90 mmol/L; 95% CI: −1.90 to 0.10; *p* = 0.08) (Table 4), though this did not reach statistical significance. However, in the context of retinopathy, males exhibited significantly lower HDL‐C levels than females (mean difference: −1.43 mmol/L; 95% CI: −2.20 to −0.66; *p* = 0.004) (Table 4). Additionally, females with retinopathy displayed higher LDL‐C levels compared to males (mean difference: −0.77 mmol/L; 95% CI: −1.55 to 0.01; *p* = 0.05) (Table 4), a difference that was marginally significant (Table 4).

These findings underscore sex‐specific variations in lipid indices among T2DM patients with complications. Specifically, reduced HDL‐C levels in males with retinopathy may reflect an altered cardiovascular risk profile. These differences highlight the need for sex‐tailored lipid monitoring and therapeutic strategies in diabetic care. Future investigations should further explore the mechanisms driving these disparities and their implications for complication risk stratification.

## 4 Discussion

This study aimed to investigate the relationship between diabetic complications and markers of oxidative stress in relation to sex among individuals with T2DM in the Ghanaian population. The findings revealed notable sex disparities in glycemic and oxidative stress markers, emphasizing the importance of considering sex‐specific factors in understanding the pathogenesis and management of diabetic complications.

Although not the primary focus of our analysis, the demographic data suggest that most participants had BMIs in the overweight range and were on standard antidiabetic therapy, primarily metformin. These baseline factors may influence oxidative stress status and should be considered in larger follow‐up studies (Table 1).

Regarding the association between sex and diabetic complications, the results showed no statistically significant differences in the prevalence of DKA, neuropathy, nephropathy, and retinopathy between males and females with T2DM (Table 2). However, notable sex disparities emerged in glycemic and oxidative stress markers (Table 3).

Males with DKA exhibited higher MDA levels compared to females, while females with retinopathy had lower MDA levels than males (Table 3). Additionally, males with DKA showed higher GSH‐Px levels than females, while no significant differences were observed between sexes without complications (Table 3). Males with DKA and retinopathy tended to have lower HDL‐C levels compared to females (Table 4), which may contribute to the increased risks of cardiovascular disease in males with T2DM.

These findings suggest that sex‐specific mechanisms may contribute to the pathogenesis and progression of diabetes complications.

One of the central findings of this study was the observation of sex‐specific differences in both MDA and GSH‐Px activity, particularly among individuals with DKA and retinopathy. Interpreting GSH‐Px within the oxidative stress framework of T2DM requires nuance, as elevated enzyme activity may either reflect a compensatory antioxidant response to increased lipid peroxidation or a more robust baseline antioxidant capacity. In our cohort, males with DKA exhibited significantly higher levels of both MDA and GSH‐Px compared to females, suggesting a redox profile marked by increased oxidative burden with parallel antioxidant mobilization. Conversely, the concurrently lower MDA and GSH‐Px levels observed in females may indicate either reduced oxidative insult or impaired enzymatic upregulation.

This dual‐marker pattern aligns with previous observations that oxidative stress and antioxidant responses in diabetes are highly context‐dependent and influenced by multiple factors, including disease severity and hormonal status [23, 24]. Estrogen, for example, has been shown to regulate both lipid peroxidation and glutathione enzyme activity, potentially affording females a transient protective advantage. These findings underscore the complexity of interpreting redox indices in isolation and highlight the need for broader biomarker panels and sex‐stratified analyses in future studies of oxidative stress in T2DM.

Previous studies have shown that sex hormones, particularly estrogen, may influence lipid metabolism and oxidative stress in diabetes. Estrogen has been shown to have protective effects against oxidative stress and inflammation, which may explain the lower MDA levels observed in females with retinopathy [8, 25]. On the other hand, although androgens play essential physiological roles in both sexes, elevated androgen levels, especially in females, have been linked to impaired insulin sensitivity and dyslipidemia, thereby increasing the risk of T2DM and related complications [25, 26].

However, a study by Liu et al. [27] found no significant differences in MDA and GSH‐Px levels between male and female T2DM patients with nephropathy, which is in contrast with the findings of the present study. While specific studies directly correlating SNP variations with sex differences in T2DM outcomes and complications were not identified, research indicates that genetic factors influencing lipid metabolism such as KLF14 and APOA1 genes can have unique clinical implications depending on the sex of the individual, necessitating a tailored approach to risk assessment and management in T2DM patients [28]. These discrepancies may be due to differences in study populations, sample sizes, and methods of measuring oxidative stress and lipid metabolism.

The observed differences between our findings and those reported by Liu et al. [27] and Alanazi et al. [28] may be partially attributable to population‐specific influences. Our study population, based in northern Ghana, may differ from those in East Asia or Europe with respect to genetic background, dietary patterns, and access to healthcare services. For example, limited intake of antioxidant‐rich foods, higher prevalence of metabolic syndrome, and delays in complication screening and management may exacerbate oxidative stress and lipid dysregulation in this population [2, 29]. In addition, socioeconomic and infrastructural disparities in resource‐limited settings can affect glycemic control and health‐seeking behavior, potentially contributing to sex‐specific variation in biomarker profiles [30]. These contextual differences highlight the importance of conducting region‐specific investigations and underscore the need for cautious interpretation of cross‐population comparisons.

The findings also suggest that sex‐specific interventions may be necessary for the management of T2DM and its complications. For example, sex hormone replacement therapy may be beneficial in reducing the risk of diabetic complications in postmenopausal women with T2DM [31]. Additionally, lifestyle interventions such as diet and exercise may have different effects on glycemic and oxidative stress markers in males and females with T2DM [24].

Furthermore, the findings highlight the importance of considering sex‐specific factors in the design and interpretation of clinical trials for T2DM and its complications. Previous studies have shown that sex differences in the response to pharmacological interventions for T2DM may exist [31]. Therefore, sex‐specific subgroup analyses should be conducted in clinical trials to ensure that the benefits and risks of interventions are accurately assessed in both males and females [32]. In addition to sex hormones, other factors such as genetics, epigenetics, and environmental exposures may also contribute to the sex disparities in T2DM and its complications [24]. Crucially, the associations between genetic variations in the KLF14, APOA1 genes and HDL‐C levels exhibit discernible sex‐specific differences [28]. Studies have suggested that male and female T2DM patients may respond differently to changes in lipid metabolism due to distinct hormonal environments and genetic backgrounds [28]. Therefore, further research is needed to elucidate the underlying mechanisms of sex disparities in T2DM and its complications and to develop sex‐tailored interventions for better management of the disease.

While most individuals were on standard therapies such as metformin or glimepiride, the effects of drug class, dose, and treatment duration on oxidative stress markers were not controlled for in the present analysis. Notably, metformin possesses antioxidant properties and may influence redox markers independent of glycemic control [33, 34]. Although the proportion of untreated participants was small (3.6%), residual confounding cannot be ruled out.

Another limitation relates to the absence of menopausal status assessment among female participants. Although women in this study ranged from 20 to 55 years, with most under age 50, we did not document whether participants were premenopausal, perimenopausal, or postmenopausal. Given that estrogen is a key modulator of antioxidant defenses, regulating GSH‐Px activity and suppressing lipid peroxidation, hormonal variation may have contributed to inter‐individual differences in oxidative stress markers. As such, some of the observed sex differences may reflect underlying hormonal status rather than diabetes‐specific effects alone.

While hypertension, a common T2DM‐related complication, was documented in our dataset, it was not included in the adjusted models. Other oxidative stress‐related comorbidities, such as cardiovascular disease or sleep apnea, were not systematically assessed.

However, the relatively young age range (20–55 years), outpatient recruitment setting, and absence of critical illness likely mitigated the influence of unmeasured comorbid conditions. Still, residual confounding cannot be excluded.

Future studies should incorporate more granular stratification and multivariable adjustment to enhance mechanistic insights into oxidative stress in T2DM. Specifically, analyses should account for pharmacotherapy class, dose, and duration; menopausal status or direct hormonal measurements; and detailed comorbidity profiling beyond core diabetes‐related complications. These refinements will help delineate the independent and interactive contributions of sex, treatment, and comorbid conditions to redox dysregulation in type 2 diabetes, ultimately informing more personalized clinical management strategies.

Our findings highlight the importance of considering sex‐specific factors in understanding the pathogenesis and management of diabetic complications. Further studies are needed to elucidate the underlying mechanisms of sex disparities in glycemic and oxidative stress markers and to develop sex‐tailored interventions for better management of T2DM and its complications. Clinical trials for T2DM and its complications should also consider sex‐specific factors in the design and interpretation of their studies.

## 5 Conclusion

While no significant differences in complication prevalence were observed between sexes, notable disparities emerged in glycemic and oxidative stress markers. Specifically, males with DKA displayed higher levels of MDA compared to females, while females with retinopathy exhibited lower MDA levels than males. Furthermore, males with DKA showed higher GSH‐Px levels than females. These findings underscore the importance of considering sex‐specific factors in the pathogenesis and management of diabetic complications, highlighting the need for further research to elucidate the underlying mechanisms driving these sex‐based differences and their implications for personalized diabetes care strategies.

## Author Contributions

Titus Naa Yeng conceived the study and led the data analysis and manuscript drafting. All authors contributed to the study design, data interpretation, and critical revision of the manuscript. All authors have read and approved the final version. The corresponding author, Titus Naa Yeng, had full access to all of the data in this study and takes full responsibility for the integrity of the data and the accuracy of the data analysis.

## Funding

The authors received no specific funding for this work.

## Disclosure

The authors declare that no funding bodies or financial relationships had any role in the design of the study; the collection, analysis, and interpretation of data; the writing of the manuscript; or the decision to submit the work for publication.

## Conflicts of Interest

The authors declare no conflicts of interest.

## Transparency Statement

The lead author, Titus Naa Yeng, affirms that this manuscript is an honest, accurate, and transparent account of the study being reported; that no important aspects of the study have been omitted; and that any discrepancies from the study as planned (and, if relevant, registered) have been explained.

## Data Availability

The data that support the findings of this study are available from the corresponding author upon reasonable request.
